# Association Between Daytime vs Overnight Digit Replantation and Surgical Outcomes

**DOI:** 10.1001/jamanetworkopen.2022.29526

**Published:** 2022-09-01

**Authors:** I-Chun F. Lin, Alfred P. Yoon, Lingxuan Kong, Lu Wang, Kevin C. Chung

**Affiliations:** 1 Section of Plastic Surgery, Department of Surgery, University of Michigan Medical School, Ann Arbor; 2 School of Public Health, Department of Biostatistics, University of Michigan, Ann Arbor

## Abstract

**Importance:**

Recent evidence suggests that select delayed replantation may not adversely affect digit survival; however, whether surgical timing (overnight or daytime) is associated with digit replantation outcomes is unknown.

**Objective:**

To assess whether digit survival, complication rate, and duration of surgery are associated with time of replantation.

**Design, Setting, and Participants:**

This retrospective case series study included all replantations performed at a single tertiary referral academic center between January 1, 2000, and August 1, 2021. Data were analyzed between October 2, 2021, and January 1, 2022. Four daytime surgery intervals were selected based on literature review. Daytime replantations started within the intervals whereas overnight replantations began outside the intervals. For each case, the procedure difficulty score and the attending surgeon expertise score were calculated. Logistic and linear regressions adjusting for confounders including procedure difficulty score and expertise score were used to assess surgical timing and outcomes. Participants were adults (aged ≥18 years) undergoing digit replantations between January 2000 and August 2021 with at least 1-month follow-up. Replantation was defined as the reattachment of a completely amputated digit that necessitated anastomosis of both artery and vein.

**Exposures:**

Daytime or overnight digit replantation.

**Main Outcomes and Measures:**

Viable replanted digit at 1-month follow-up, number of complications, and duration of surgery.

**Results:**

A total of 98 patients (mean [SD] age, 39.5 [15.3] years; 136 [93%] men) and 147 digits met inclusion criteria. Overall success rate was 55%. Between 4 pm and 7 am, overnight replantations were associated with 0.4 fewer complications (β, −0.4; 95% CI, −0.8 to −0.1) and 90.7 minutes shorter operative time (β, −90.7; 95% CI, −173.6 to −7.7). A 1-point increase in surgeon expertise score was associated with 1.7 times increased odds of replantation success for all intervals (adjusted odds ratio, 1.7; 95% CI, 1.2 to 2.4; *P* = .002). There were no differences in digit survival by surgical time.

**Conclusions and Relevance:**

In this case series study of digit replantations, time of operation was not associated with replantation success. Overnight replantation was associated with fewer complications and shorter duration of surgery compared with daytime surgery. Results of this study suggest that overnight replantations may be performed with outcomes comparable to daytime replantations at a tertiary care academic center.

## Key Points

**Question:**

Is time of operation (daytime vs overnight) associated with digit replantation outcomes?

**Findings:**

In this single-center case series including 98 patients and 147 digits, digit survival was not associated with time of operation. Overnight replantations were associated with fewer complications and shorter duration of surgery compared with daytime replantations.

**Meaning:**

This study supports the safety of performing overnight replantations at a large academic center and suggests that replantation outcomes are similar whether surgery is performed during the day or overnight.

## Teaser

This case series study at a tertiary care academic center assesses whether surgical time, categorized as either daytime or overnight, is associated with digit replantation outcomes.

## Introduction

Approximately 5000 digit replantations are performed yearly in the US. Many of these procedures are performed overnight, usually after delayed arrival at a replantation center after multiple interfacility transfers. These overnight digit replantations have been perceived as a necessity to maximize the probability of digit survival constrained by a critical ischemic window.^1,2,3,4,5^ However, many studies contest the importance of minimizing ischemia time given the substantial heterogeneity in assessment and outcome data.^6,7,8,9,10,11,12,13^ Digits lack muscle; therefore, ischemia time can be prolonged with appropriate cooling. Furthermore, sleep deprivation and fatigue have been associated with deficits in attention, memory, and decision-making, as well as an increase in risk-taking behaviors.^14,15^ These performance decreases may translate to a need for surgery, although previous studies suggested the deleterious outcomes associated with fatigue can be attenuated by surgeon skill and expertise.^16,17,18,19^

Studies from Europe and Asia have reported the possibility of delaying select overnight replantations.^6,20,21^ Cavadas et al^20^ reported that amputated digits with less than 12 hours of cold ischemia presenting after 6 pm can undergo delayed replantation in the morning. Ischemia-sensitive replants (transmetacarpal, crush or contaminated, or extended ischemia times) were performed immediately. Woo et al^21^ reported a similar protocol that delayed single-digit amputations until the following morning at 7 am. No significant difference in digit survival was observed in either study between delayed and immediate replantations.^20,21^ This possibility of reducing overnight burden may be appealing to hand surgeons. Although there is limited evidence that replantations can be delayed safely, it remains unknown whether daytime or overnight replantations have different outcomes because of surgeon factors. Studies in other surgical specialties reported associations between overnight operations and worse outcomes, with conflicting results.^14,15,16,17,19,22,23,24,25,26,27,28,29,30,31,32,33,34,35,36,37,38,39,40,41,42^ Only 1 study to date has investigated digit replantation start time and its association with outcomes.^26^ In this study, the authors found that replantations performed between 8 am and 6 pm had significantly better survival than those performed outside that window. However, these findings may not be generalizable to other health care settings.

We conducted a retrospective case series study at a tertiary care academic center to assess surgical time, categorized as either daytime or overnight, and replantation outcomes. We investigated surgical timing and replantation outcomes using previously described time intervals^20,22,26,27,28,29,30,32,37,41,43,44,45,46,47,48,49^ and adjusted for both procedure difficulty and surgeon skill.^50^ We hypothesized that there would be no difference in digit survival, complication rates, and surgery duration between daytime and overnight digit replantations.

## Methods

### Study Population and Data Source

All adults (aged 18 years or older) who were taken to the operating room for attempted replantation between January 2000 and August 2021 were identified through the University of Michigan electronic medical records using *International Classification of Diseases, Ninth Revision* codes (codes 84.21 and 84.22) and *Current Procedural Terminology* codes (codes 20827, 20824, 20816, 20822, and 35207). Only those digits that were amenable to replantation at the time of injury as documented in the operative report by the surgeon with at least 1-month follow-up were included (Figure). Patients who intended to undergo replantation but instead required revision amputation during the initial surgery were excluded with the assumption that replantation was not technically feasible. Data on race and ethnicity were not collected for analysis. This study was conducted between September 1, 2021, and October 1, 2021; data analysis was conducted between October 2, 2021, and January 1, 2022. We adhered to the reporting guideline for case series. This study was considered exempt from regulation and informed consent requirements by the University of Michigan institutional review board because of anonymized secondary use of identifiable data.

**Figure.**
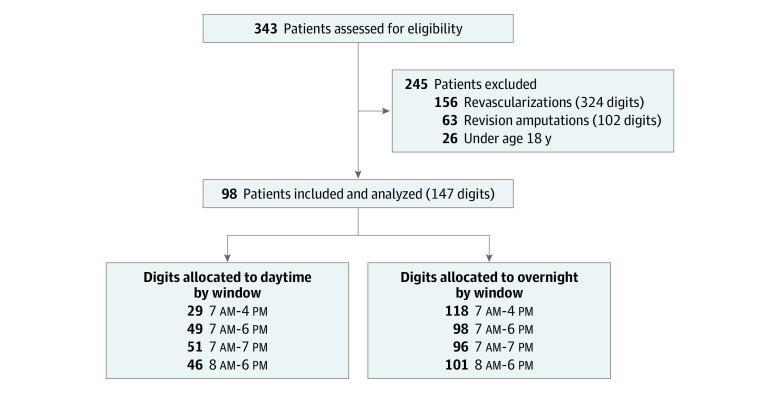
Patient and Digit Flow Diagram

### Variables and Outcomes

The following patient and injury characteristics were collected from the electronic medical records: age, sex, smoking status (former, no, unknown, or yes), Elixhauser Comorbidity Index score (range, −19 to 89, with a higher score indicating higher likelihood of in-hospital mortality), affected digit (thumb vs other), number of affected digits, mechanism of injury (sharp, crush, or avulsion), zone of injury, and ischemia time. Operative characteristics included surgery start and end times, number of replanted digits per case, number of anastomosed arteries and veins, vein graft use, procedure difficulty score, and attending surgeon expertise score. Replantation was defined as the reattachment of a completely amputated digit that necessitated anastomosis of both artery and vein. A procedure difficulty score (cohort range, 1.5-15.8 [theoretical range, 1.5-20.8] with a higher score indicating higher procedure difficulty), determined by smoking status, zone of injury, multidigit or single-digit amputation, and mechanism of injury,^50^ was calculated for each replanted digit. Surgeon expertise score^50^ (range, 1.26-5.71 with larger values indicating a higher surgeon proficiency with replantation), determined by average case difficulty score of successful replantations adjusted by the inverse of the average case difficulty score of failed replantations divided by total procedure volume, was calculated for each attending surgeon. Postoperative complications (stiffness, nonunion, severe infections, and revision surgeries) were collected from the electronic medical record through operative reports and clinic notes. The primary outcome of this study was replantation success, defined as a viable replanted digit at 1-month follow-up. Secondary outcomes included complications and duration of surgery.

### Daytime vs Overnight Replantations

After literature review, we selected the 4 most commonly defined daytime surgery intervals: 7 am to 4 pm, 7 am to 6 pm, 7 am to 7 pm, and 8 am to 6 pm. Procedures were categorized by surgical start time.^22,23,42^ Daytime procedures began within the stated time intervals, whereas overnight procedures began outside the intervals.

### Statistical Analysis

Multiple linear regression models were created with the following variables of interest to assess total number of complications and duration of surgery: age, sex, Elixhauser Comorbidity Index score, number of replanted digits, ischemia time, number of arterial and venous anastomoses, surgeon expertise score, and procedure difficulty score. Logistic regression models were created with the following variables of interest to determine their effects on replantation success: age, sex, ischemia time, number of venous anastomoses, and surgeon expertise score. The unit of analysis was each digit, as a patient could have sustained multiple digit amputations and each digit may have had different outcomes. The *t* test was used for continuous outcomes; χ^2^ test or Fisher exact methods were used for categorical outcomes. A 2-tailed α of .05 was the criterion for significance. Statistical analyses were performed by an independent statistician (L.K.) using R version 4.1.2 (R Foundation for Statistical Computing).

A post hoc power analysis was conducted using the sample size n = 147 and α = .05. Analyses were performed under the assumption that our models were correct and included all possible confounders. For the linear models, we achieved 0.54 power for total complications and 0.99 power for duration of surgery. For the logistic regression model, we assumed that surgery time follows a Poisson distribution and achieved 0.74 power for replantation success. Power analyses were carried out with GPower statistical software version 3.1.9.7.

## Results

A total of 98 patients and 147 replanted digits were included in the analysis (Table 1). Of the replantations performed, 93% (136) were from male patients and 7% (11) were from female patients, with a mean (SD) age of 39.5 (15.3) years. Multidigit replantations accounted for 56% (82 digits from 33 patients) of the replantations performed. The thumb was the most replanted digit (34% [50 of 147]), followed by the middle finger (26% [38 of 147]) then the ring finger (17% [25 of 147]), index finger (16% [23 of 147]), and small finger (7% [11 of 147]). The mean (SD) procedure difficulty score was 6.0 (3.8) (range, 1.5-20.8), whereas the mean (SD) surgeon expertise score was 3.6 (1.3) (range, 1.0-5.7). The overall replantation success rate was 55% (n = 81).

**Table 1.** Descriptive Statistics of Patient Cohort by Digit

Characteristic	Patients, mean (SD)
Age, y	39.5 (15.3)
Sex, No. (%)	
Male	136 (93)
Female	11 (7)
Smoking status, No. (%)	
Former	17 (12)
No	61 (39)
Unknown	11 (7)
Yes	58 (39)
Elixhauser Comorbidity Index score^a^	0.8 (1.3)
Ischemia time, min	418 (129)
Digits replanted per case	2 (1)
No. of arteries anastomosed	1 (0.4)
No. of veins anastomosed	1 (0.9)
Total No. of complications	1.1 (0.8)
Procedure difficulty score^b^	6.0 (3.8)
Failed digits, No. (%)	66 (45)
Length of stay, d	8.2 (4.8)
Duration of surgery, min	465 (211)
Length of follow-up, mo	8.6 (8.3)

^a^ Elixhauser Comorbidity Index score with a cohort range of 0 to 7, higher score indicating higher likelihood of in-hospital mortality; the theoretical range is −19 to 89.

^b^ Procedure difficulty score (as reported in Yoon et al^50^): cohort range was 1.5 to 15.8, with a higher score indicating higher procedure difficulty. Calculated using pooled relative risk (RR) of injury characteristics, as follows: replantation vs revascularization (pooled RR, 1.5), active smoker vs nonsmoker (pooled RR, 2.3), multiple digits per case vs single digit (pooled RR, 1.8), crush vs sharp injury (pooled RR, 2.0), avulsion vs sharp injury (pooled RR, 2.5), and distal zone of injury (pooled RR, 1.3). Theoretical range was 1.5 to 20.8, most difficult case scenario [replantation + active smoking status + multiple digits per case + avulsion injury + distal zone].

Daytime and overnight cohorts did not differ significantly in all patient characteristics, although there was a slightly higher male predominance overnight (94 [96%] vs 42 [86%]; *P* = .04) (Table 2). When daytime was defined as 7 am to 4 pm, overnight replantations were associated with 0.4 fewer complications (β, −0.4; 95% CI, −0.8 to −0.1) and 90.7 minutes shorter operative time (β, −90.7; 95% CI, −173.6 to −7.7) (Table 3) (eTable in the Supplement). A 1-point increase in surgeon expertise score was associated with 1.7 times increased odds of replantation success regardless of the intervals defined for daytime surgery (adjusted odds ratio, 1.7; 95% CI, 1.2 to 2.4; *P* = .002). Surgical time was not significantly associated with replantation success.

**Table 2.** Daytime vs Overnight Cohort Characteristics^a^

Characteristics	Digits, No. (%)		*P* value^b^
	Daytime (n = 49)	Overnight (n = 98)	
Age, mean (SD), y	37.9 (14.6)	40.3 (15.7)	.36
Sex			
Male	42 (86)	94 (96)	.04
Female	7 (14)	4 (4)	
Smoking status			
Current	21 (43)	37 (38)	.61
Former	4 (8)	13 (13)	
Never	19 (39)	42 (43)	
Unknown	5 (10)	6 (6)	
Elixhauser Comorbidity Index score^c^			
0	24 (49)	55 (56)	.06
1	11 (22)	29 (30)	
2	10 (20)	5 (5)	
3	2 (4)	2 (2)	
4	1 (2)	4 (4)	
≥5	0	3 (3)	
Ischemia time, h			
<12	47 (96)	80 (82)	.55
≥12	0	3 (3)	
No. of replanted digits per case			
1	19 (39)	46 (47)	.38
≥2	30 (61)	52 (53)	
No. of arteries anastomosed			
1	44 (90)	79 (81)	.45
2	5 (10)	15 (15)	
No. of veins anastomosed			
0	4 (8)	11 (11)	.13
1	22 (45)	56 (57)	
2	20 (41)	22 (22)	
3	2 (4)	2 (2)	
4	1 (2)	7 (7)	
Procedure difficulty score, mean (SD)	5.2 (2.9)	6.4 (4.1)	.04
Surgeon expertise score, mean (SD)	3.1 (1.3)	2.8 (1.6)	.18
No. of failed digits			
Yes	17 (35)	49 (50)	.11
No	32 (65)	49 (50)	
Length of stay, mean (SD), d	7.6 (3.5)	8.6 (5.3)	.20
Duration of surgery, mean (SD), min	518 (225)	438 (200)	.04

^a^ Daytime defined as cases that start between 7 am and 6 pm for this cohort. Unadjusted results. Frequencies may not sum to 100% because of missing values.

^b^ The *t* test for continuous outcomes; χ^2^ test or Fisher exact methods for categorical outcomes.

^c^ Elixhauser Comorbidity Index score with a cohort range of 0 to 7, with a higher score indicating higher likelihood of in-hospital mortality; the theoretical range is −19 to 89.

**Table 3.** Multivariable Regression Results for Daytime and Overnight Replantation

Outcome	Daytime surgery between 7 am and 4 pm (n = 29)		Daytime surgery between 7 am and 6 pm (n = 49)		Daytime surgery between 7 am and 7 pm (n = 51)		Daytime surgery between 8 am and 6 pm (n = 46)	
	β (95% CI)	*P* value	β (95% CI)	*P* value	β (95% CI)	*P* value	β (95% CI)	*P* value
**Replantation success^a^**								
Overnight vs daytime replant, aOR (95% CI)	0.7 (0.3 to 2.0)	.55	0.5 (0.2 to 1.1)	.11	0.6 (0.3 to 1.3)	.19	0.4 (0.2 to 1.1)	.08
Ischemia time, min, aOR (95% CI)	1.0 (1.0 to 1.0)	.02	1.0 (1.0 to 1.0)	.02	1.0 (1.0 to 1.0)	.02	1.0 (1.0 to 1.0)	.02
Vein number, aOR (95% CI)	0.9 (0.6 to 1.3)	.52	0.8 (0.5 to 1.3)	.47	0.9 (0.5 to 1.3)	.50	0.8 (0.5 to 1.3)	.44
Surgeon expertise score, aOR (95% CI)	1.6 (1.2 to 2.2)	<.01	1.6 (1.2 to 2.2)	<.01	1.6 (1.2 to 2.2)	<.01	1.6 (1.2 to 2.2)	<.01
**Total complications^b^**								
Overnight vs daytime replant	−0.4 (−0.8 to −0.1)	.02	−0.1 (−0.4 to 0.2)	.39	−0.2 (−0.4 to 0.1)	.27	0.1 (−0.2 to 0.4)	.47
No. of digits per case	−0.1 (−0.3 to 0.0)	.12	−0.1 (−0.3 to 0.0)	.05	−0.1 (−0.3 to 0.0)	.05	−0.1 (−0.3 to 0.0)	.05
Duration of surgery^b^								
Overnight vs daytime replant	−90.7 (−173.6 to −7.7)	.03	−58.6 (−124.2 to 6.9)	.08	−45.6 (−109.9 to 18.7)	.16	−63.3 (−133.0 to 6.4)	.07
Sex (female vs male)	169.1 (24.4 to 313.9)	.02	207.6 (72.7 to 342.5)	<.01	215.5 (80.4 to 350.7)	<.01	200.5 (63.5 to 337.6)	<.01
No. of digits per case	79.7 (47.4 to 112.0)	<.01	72.1 (40.2 to 104.1)	<.01	73.3 (41.2 to 105.4)	<.01	72.2 (40.2 to 104.1)	<.01

Abbreviation: aOR, adjusted odds ratio.

^a^ Logistic regression formula: log {*P* (replant success)/[1 − *P* (replant success)]} = day or night surgery + age + sex + ischemia time + number of anastomosed veins + surgeon expertise score.

^b^ Linear regression formula: outcome (total complications, length of stay, duration of surgery) = day or night surgery + age + sex + Elixhauser score + number of replanted digits + ischemia time + number of anastomosed arteries + number of anastomosed veins + procedure difficulty score + surgeon expertise score + ε; ε denotes a random error follows normal distribution.

## Discussion

In this case series study, we observed that overnight or daytime surgery was not associated with replantation success rates. Overnight replantations were associated with fewer complications and shorter operative times, but this result was contingent on the time interval defined as daytime surgery. Our results seem to conflict with those of previous studies that found daytime operations were associated with better surgical outcomes.

Notably, our outcomes varied with the time intervals used to define daytime surgery. When setting 7 am to 4 pm as the daytime interval, overnight replantations were associated with fewer complications and shorter operative times. Previous studies have suggested that overnight procedures precipitate longer duration of surgery,^29,30^ which may be associated with limited staff and overnight surgical teams that are unfamiliar with the procedures, compromising surgical outcomes. It may be that replantations performed overnight imbue a sense of urgency or that surgeons performing overnight replantations work more efficiently to shorten operative time to minimize fatigue that may compromise surgical outcomes.

It is difficult to explain why overnight replantations were associated with decreased complications compared with daytime replantations. It could be that shorter duration of surgery leads to fewer complications, as previous studies have suggested longer operative times are associated with more complications.^51,52^ However, only 1 of the 4 daytime intervals found differences in complication rates and surgical duration; therefore, these results should be interpreted with caution.

Our findings differ from those of Breahna et al,^26^ which reported increased odds of replantation success when performed between 8 am and 6 pm compared with outside that interval. Digit survival was the only outcome measure assessed in that study, but it was unclear whether digit survival was assessed at discharge or after a certain follow-up time to account for late failures. Because replantations can fail even after a 1-week postoperative period,^53^ the study’s findings may have differed if digit survival had been assessed after a longer follow-up. The authors used surgeon rank (either consultant plastic surgeon or a senior hand fellow) as a surrogate measure for surgeon skill. However, because skill varies widely even among experienced surgeons,^50^ it is plausible that the differences in their outcomes may be associated with variation in surgeon expertise. This plausibility is evident in our logistic regression results for replantation success, which identified a significant association between higher surgeon expertise score and replantation success even when adjusting for surgical timing. Nevertheless, the authors attributed their findings to the availability of both a senior hand surgeon and a full complement of rested operating room staff during work hours. Investigations of general surgery and orthopedic surgery procedures have also credited the difference in staffing, fatigue, and inadequate oversight to worse outcomes after overnight surgery.^14,15,16,17,19,22,23,24,25,26,27,28,54,55^ In a large tertiary academic center that participates in the American Society for Surgery of the Hand/American College of Surgeons National Hand Trauma Center Network^56^ such as ours (Michigan Medicine), overnight call can be appropriately distributed among surgeons thereby alleviating the cumulative burden of fatigue which may more heavily weigh on smaller centers without such resources. For example, a single hand surgeon at a smaller center or private practice may be responsible for most of the clinic’s replantations^20^ whereas hand call is split among 7 attending surgeons at our center. This distribution is supported by a study conducted at a similar tertiary academic center which found no differences in operative morbidity and mortality between daytime and overnight general surgery procedures.^28^ Previous evidence suggests experienced surgeons may be able to compensate for the deleterious effects of overnight surgery, making up to 25% fewer errors than resident surgeons even under fatigue.^18^ Our findings suggest that factors commonly ascribed to poor outcomes after overnight surgeries may be offset by a larger pool of well-trained surgeons and staff familiar with replantations.

Unlike some general surgery and orthopedic procedures, digit replantations are not considered elective; there is a finite ischemic time window within which success rates are higher.^1,2,3,4,5^ Furthermore, tertiary referral centers often do not have the ability to choose to delay replantations because patients frequently arrive after an extensive referral process between hospitals, which may compromise their critical ischemic window. Because of this factor, the surgeons at our institution do not delay replantations. From a health care systems perspective, surgeons frequently incur significant opportunity cost when canceling daytime elective procedures for a replantation case because of limited operative block time availability. A tangible solution to encourage and facilitate daytime replantations is a flexible operative block time that enables hand surgeons to delay elective cases to the following day for an urgent replantation. Our findings suggest that replantation surgeons at high-volume tertiary referral centers able to compensate for fatigue, producing similar replantation outcomes whether performed overnight or during the day. Centers in which surgeons encounter high replantation volumes may demonstrate similar consistency in outcomes. Our results support that surgeon expertise, rather than the time of surgery, is the important factor contributing to replantation success. Although there may be no benefit to preferentially performing replantations during the day vs overnight, there is also no detriment in performing replantations in either window. Therefore, if the patient presents overnight within the critical ischemic window or the amputated segments can be stored in a manner that will prolong that window, delaying replantation until daytime is feasible but likely will not confer any improvements in outcome compared with overnight replantations.

### Limitations

This study has limitations. First, this study was a single-center study conducted at a large tertiary academic center; therefore, the results may not be generalizable to smaller centers with fewer overnight resources. But our findings may be applicable to other tertiary referral centers that commonly perform digit replantations. Another limitation was our modest sample size compared with studies investigating overnight procedures in other surgical specialties.^22,23,28^ However, statistical power analysis revealed that 2 of 3 outcomes (replantation success and duration of surgery) either had sufficient or near sufficient power to draw meaningful conclusions. In addition, our sample size is comparable to other digit replantation studies conducted in the US and is larger compared with the only published study to date investigating overnight replantations.^26^ Furthermore, our replantation success rate is similar to that of other tertiary academic centers in the US.^57,58^ Nevertheless, the results of this study should be validated by research from other institutions. We chose to extract data from the electronic medical record at our institution as opposed to a larger administrative data set, knowing that a tradeoff for high granularity of data would be loss of sample size. Even so, the ability of our multivariable regression to adjust for confounding factors was limited to certain patient and operation characteristics, and differences in outcomes identified in our investigation may be attributable to other perioperative factors such as surgical team dynamics and postoperative care. Some replantation complications may develop over longer periods of time beyond the follow-up length of this study; therefore, true complication rates may be higher than that in our study. Next, we selected daytime intervals based on a thorough literature review. In contrast, many studies defined their windows using institution-specific surgical scheduling practices or work hours. Given the emergent nature of digit replantations, this definition was not feasible for our study. Similarly, we recognize that using other time intervals may identify additional differences in outcomes. In addition, the goal of this study was not to compare delayed vs immediate replantations; rather, this study assessed whether performing replants during the day vs overnight was associated with outcomes.

## Conclusions

In this study, overnight replantations were not associated with increased replantation failure compared with daytime replantations at a tertiary care hospital. Overnight replantations were associated with decreased number of complications and shorter surgical duration. These associations were present even after controlling for patient, injury, and surgeon characteristics. This study supports the safety of performing overnight replantations at a large academic center and suggests that replantation outcomes are similar whether performed during the day or overnight.

## Notes

Supplement. **eTable.** Complications by Cohort (Daytime Versus Overnight)
