# Supplementary material for: Association Between Daytime vs Overnight Digit Replantation and Surgical Outcomes
Source: JAMA Netw Open. 2022 Sep 1;5(9):e2229526. doi: 10.1001/jamanetworkopen.2022.29526 (PMC9437749; doi:10.1001/jamanetworkopen.2022.29526)
Supplement: Supplement. — eTable. Complications by Cohort (Daytime Versus Overnight) [file jamanetwopen-e2229526-s001.pdf]

Supplemental Online Content

Lin ICF, Yoon AP, Kong L, Wang L, Chung KC. Association between daytime vs overnight digit replantation and surgical outcomes. *JAMA Netw Open*. 2022;5(9):e2229526. doi:10.1001/jamanetworkopen.2022.29526

eTable. Complications by Cohort (Daytime Versus Overnight)

This supplemental material has been provided by the authors to give readers additional information about their work.

© 2022 Lin ICF et al. *JAMA Network Open*.

eTable. Complications by Cohort (Daytime Versus Overnight)

		Digits, No. (%)							
		7AM to 4PM[a]		7AM to 6PM[b]		7AM to 7PM[c]		8AM to 6PM[d]	
	**Overall**	Daytime	Overnight	Daytime	Overnight	Daytime	Overnight	Daytime	Overnight
Complications, requiring revision surgery (excluding revision amputation)[e]									
0	141 (69)	22 (76)	79 (67)	41 (84)	60 (61)	41 (80)	60 (62)	41 (89)	60 (59)
1	37 (25)	3 (10)	34 (29)	4 (8)	33 (34)	6 (12)	31 (33)	2 (4)	35 (35)
> 1	9 (6)	4 (14)	5 (4)	4 (8)	5 (5)	4 (8)	5 (5)	3 (7)	6 (6)
Complications, non-surgical[f]									
0	114 (78)	17 (59)	97 (76)	37 (76)	77 (79)	39 (76)	75 (78)	39 (85)	76 (75)
1	30 (20)	10 (34)	20 (17)	10 (20)	20 (20)	10 (20)	20 (21)	5 (11)	24 (24)
> 1	3 (2)	2 (7)	1 (1)	2 (4)	1 (1)	2 (4)	1 (1)	2 (4)	1 (1)

[a] Daytime cohort n = 29

[b] Daytime cohort n = 49

[c] Daytime cohort n = 51

[d] Daytime cohort n = 46

[e] Complications requiring revision surgery, including nonunion

[f] Complications not requiring revision surgery, including stiffness and severe infection

© 2022 Lin ICF et al. *JAMA Network Open*.
